# Exercise intensity and mortality in overweight and obese patients with chronic kidney disease: longitudinal analysis (1999–2016)

**DOI:** 10.1186/s12889-024-20498-6

**Published:** 2024-10-31

**Authors:** Chuyue Qian, Fengjun Zhou, Dandan Lu, Jingda Huang, Mindan Sun

**Affiliations:** Department of Nephrology, The First Hospital of Jilin University, Changchun, China

**Keywords:** Vigorous physical activity, Chronic kidney disease, Overweight, Obese, All-cause mortality

## Abstract

**Background:**

Chronic kidney disease (CKD) and overweight/obesity are significant global public health issues. Appropriate free-time physical activity (PA) is essential for overweight/obese patients with chronic kidney disease, but specific guidelines are lacking. The present study was conducted to determine the association between PA and all-cause mortality in these patients.

**Methods:**

Data from 3,434 overweight/obese adults with CKD from the 1999–2016 National Health and Nutrition Examination Surveys were analyzed. Associations between clinical/laboratory findings and PA intensity (moderate and vigorous) were investigated. The all-cause mortality of patients in different PA categories were compared by Kaplan–Meier analysis. Factors associated with all-cause mortality were determined using a Cox proportional hazards model. A restricted cubic spline was employed to obtain a more flexible and detailed representation of the relationship between PA intensity and all-cause mortality, with better predictive capability.

**Results:**

The Kaplan–Meier analysis revealed that greater all-cause mortality was associated with < 10 min/week moderate/vigorous PA (log-rank *p* < 0.001). A greater survival probability was associated with ≥ 150 min/week vigorous PA or 10–149 min/week moderate PA (log-rank *p* < 0.001). Age, gender, vigorous PA, smoking status, alcohol consumption, diabetes status, eGFR, serum albumin level, uric acid level, and blood urea nitrogen level were identified as factors associated independently with mortality in the Cox proportional hazards analysis. The restricted cubic splines revealed that these relationships were non-linear (all *p* < 0.05). Kaplan–Meier analysis of data from patients who engaged in 10–450 min/week moderate/vigorous PA revealed significant differences between the 0–74-min/week and other vigorous PA groups (all log-rank *p* < 0.001).

**Conclusions:**

Extended durations of vigorous PA are associated with reduced all-cause mortality in overweight/obese patients with CKD. Clinicians should recommend vigorous free-time PA to these patients, and public health interventions should target this goal to maximize patient health.

**Supplementary Information:**

The online version contains supplementary material available at 10.1186/s12889-024-20498-6.

## Background

Chronic kidney disease (CKD) is a serious global health issue, and a significant risk factor for cardiovascular disease and increased morbidity and mortality. It affects more than 850 million individuals worldwide, presenting a major public health challenge and adding to the social burden [1, 2]. Despite the availability of many treatments for kidney disease, the high mortality rate for patients with CKD remains unmitigated. As a result, auxiliary intervention strategies promoting changes in patients’ diet, sleep, and lifestyle that involve weight management and physical activity (PA) are being explored in clinical practice.

The global surge in obesity rates has created an additional public health crisis [3]. The relationship between the body mass index (BMI) and renal insufficiency is U-shaped and becomes stronger with age [4]. Numerous real-world studies have revealed positive correlations of the BMI with the development of proteinuria, a decline in the estimated glomerular filtration rate (eGFR), and the increased incidence of end-stage renal disease (ESRD) [5, 6]. Obesity is an independent risk factor for primary CKD progression, potentially worsening the prognosis [7]. Furthermore, it is often associated with metabolic syndrome, which can result in the development of diseases such as cardiovascular disease, diabetes, and cancer. Consequently, weight loss has been considered as a logical therapeutic approach, and is often dependent on PA.

PA is typically classified as occupational or non-occupational, the latter including household work, commuting, and free-time PA. Evidence suggests that occupational PA does not improve health, and may increase the cardiovascular disease risk and mortality outcomes [8]. Occupational PA was not associated with CKD in a cohort of 475,376 individuals in China [9]. However, there are lots of research supporting the health benefits of non-occupational PA, espicially free-time PA. These contrasting health impacts of free-time and occupational PA are embodied in the concept of the PA health paradox [10]. Holtermann et al. [11] identified six reasons for this paradox: lower PA intensity, longer PA duration, insufficient recovery time, increased inflammation, elevated 24-h heart rate and blood pressure.

Regular PA offers multiple benefits, such as the reduction of endothelial dysfunction, proatherogenic adipokine levels, systemic inflammation, insulin resistance, and visceral fat accumulation [12–14]. However, no clear PA guideline has been established specifically for overweight and obese patients with CKD. Among existing authoritative guidelines for PA, the 2020 World Health Organization (WHO) guidelines recommend that adults engage in 150–300 min moderate physical activity (MPA), 75–150 min vigorous physical activity (VPA), or an equivalent combination per week [15]. The 2024 Kidney Disease: Improving Global Outcomes (KDIGO) clinical practice guidelines recommend that patients with CKD engage in a minimum of 150 min MPA per week, or as much as their cardiovascular and physical conditions allow [2]. The 2016 American Association of Clinical Endocrinologists/American College of Endocrinology (AACE/ACE) guidelines recommend that overweight and obese individuals engage in at least 150 min MPA per week [16]. The 2019 American College of Cardiology/American Heart Association (ACC/AHA) guidelines for the primary prevention of cardiovascular disease recommend that adults engage in a minimum of 150 min MPA or 75 min VPA weekly [17]. In addition to these exercise duration recommendations, findings from a recent study highlight the mortality-reducing benefits of increasing VPA [18]. Thus, the present study was conducted to determine the association between free-time PA and all-cause mortality in overweight and obese patients with CKD, and the optimal combination of VPA and MPA for these patients.

## Methods

### Study population

The National Health and Nutrition Examination Survey (NHANES) is a comprehensive, cross-sectional study assessing of adults and children across the United States. The present study was conducted using data from the NHANES collected between 1999 and 2016. In addition, mortality data up to 2019 were obtained from the publicly available National Death Index (NDI). Detailed information about the data is provided on the NCHS/CDC website. The NHANES is administered by the National Center for Health Statistics (NCHS) of the Centers for Disease Control and Prevention (CDC), with authorization from the NCHS Institutional Review Board. All participants provided written informed consent to their involvement in the survey.

**Fig. 1:**
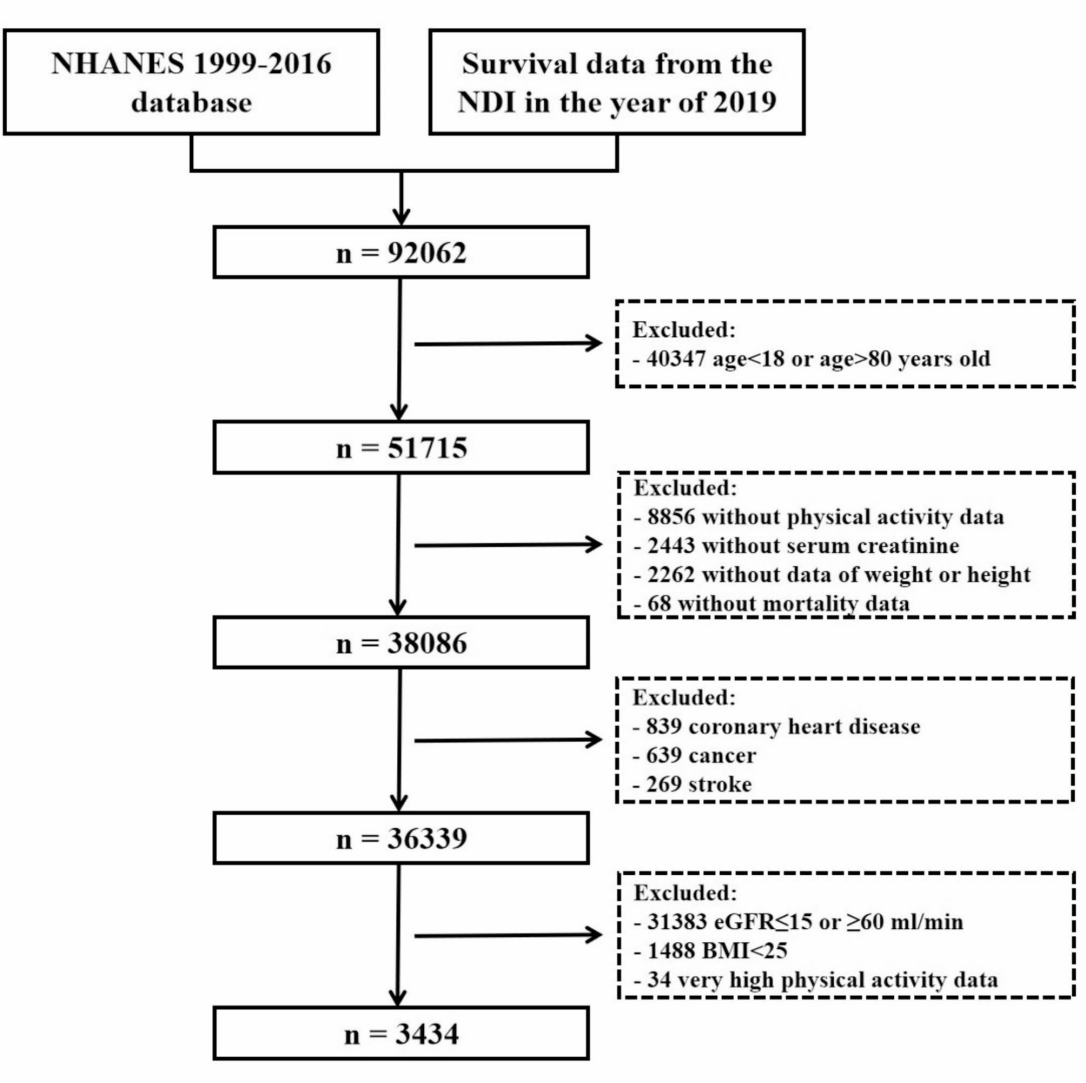
Flowchart of individuals’ inclusion in the study. NHANSE, National Health and Nutrition Examination Survey; NDI, National Death Index; eGFR, estimated glomerular filtration rate; BMI, body mass index

Data from 92,062 participants were assessed for eligibility. The exclusion criteria were: (1) age < 18 or > 80 years (*n* = 40,347); (2) lack of data on PA (*n* = 8,856), the serum creatinine level (*n* = 2,443), weight and/or height (*n* = 2,262), and/or mortality (*n* = 68); (3) diagnosis of coronary heart disease (*n* = 839), cancer (*n* = 639), or stroke (*n* = 269) at baseline; and (4) eGFR ≤ 15 or ≥ 60 mL/min/1.73 m^2^ (*n* = 31,383), BMI < 25 kg/m^2^ (*n* = 1,488), or very high PA [> 4 h moderate-to-vigorous physical activity (MVPA)/day; *n* = 34; Fig. 1]. The sample for final analysis thus comprised 3,434 individuals.

## PA definitions

PA data were obtained using the WHO-designed Global Physical Activity Questionnaire [19]. PA is usually quantified using metabolic equivalent of task (MET) values, considering the type, frequency, and duration of each weekly activity: PA (MET min/week) = MET × weekly frequency × duration [20]. However, a single recommended MET value was provided for each PA level beginning with the 2007–2008 NHANES. For the present study, MPA was defined as 3.00–5.90 METs and VPA was defined as ≥ 6.00 METs. The weekly durations of VPA and MPA were determined by multiplying the weekly frequency by the duration of each activity. VPA and MPA durations were summed to obtain MVPA durations. Subjects were then classified according to weekly VPA and MPA durations of < 10, 10–149, and ≥ 150 min. Subjects who engaged in MVPA were classified according to the durations of < 10, 10–149, 150–299, and ≥ 300 min/week. For the determination of the optimal PA duration, subjects who engaged in 10–450 min MVPA per week were further divided according to VPA durations of 0–74, 75–149, 150–224, and ≥ 225 min/week.

## Analysis of covariance

NHANES data on individuals’ age, gender, race/ethnicity, educational level, smoking, alcohol consumption, weight, height, and personal medical histories were extracted for analysis. BMIs were calculated as: weight (kg) / height (m^2^), overweight was defined as 25 ≤ BMI < 30 kg/ m^2^. obese was defined as BMI ≥ 30 kg/ m^2^. Alcohol use was classified as < 1 and ≥ 1 drink/day. Smoking was classified as < 100 and ≥ 100 cigarettes in one’s life. The baseline serum urea nitrogen, uric acid, creatinine, albumin, total cholesterol, and triglyceride levels were obtained from laboratory test results sourced from the NHANES. eGFRs were calculated using the Chronic Kidney Disease Epidemiology Collaboration formula [21]. CKD was defined as eGFR < 60 mL/min/1.73 m^2^.

### Statistical analysis

The data were analyzed using the R software (http://www.R-project.org, The R Foundation). Categorical variables are expressed as frequencies and percentages, and continuous variables are expressed as medians with interquartile ranges. The chi-squared test was used to compare two categorical variables. The Mann–Whitney U test was used to compare two continuous variables and the Kruskal–Wallis test was used to compare more than two variables. Associations of PA categories with all-cause mortality were investigated by Kaplan–Meier analysis. Univariable Cox proportional hazards analysis was used to evaluate associations of variables with all-cause mortality. Variables with p values < 0.01, including VPA, MPA, and MVPA, were included in a multivariable analysis. A restricted cubic spline model with various PA percentiles was used to explore the relationship between PA intensity (as a continuous variable) and all-cause mortality, and to assess the predictive capability of PA. p values < 0.05 were considered to be significant.

## Results

### Subject characteristics

The subjects’ baseline characteristics are presented in Table 1. The median age was 61 years, and 80.00% of the subjects were male. The median follow-up duration was 103.00 months. In total, 638 (18.58%) subjects died during the follow-up period. This group was older and less educated, and had higher prevalences of smoking, alcohol consumption, and hypertension than did subjects who survived. In addition, non-survivors had lower eGFRs and serum albumin and total cholesterol levels, and higher blood levels of urea nitrogen, uric acid, and creatinine, than did survivors. Non-survivors spent significantly less time than survivors engaging in VPA and MVPA.

**Table 1:** Baseline patient characteristics according to all-cause mortality

Variable	Total (*n* = 3,434)	Survivors (*n* = 2,796)	Non-survivors (*n* = 638)	*p*	
Clinical characteristics					
Age (years)	61 (50–71)	60.00 (48–68)	74.00 (66–80)	< 0.001	
Gender (male)	2,748 (80.00)	2,272 (81.30)	475 (74.50)	< 0.001	
Race/ethnicity				0.010	
Non-Hispanic white	1,542 (44.90)	1,186 (42.40)	356 (55.80)		
Other	1,892(55.10)	1,610 (57.60)	282 (44.20)		
Education				< 0.001	
High school graduation or less	1,605 (46.70)	1,208 (43.20)	397 (62.20)		
College graduation or more	1,829 (53.30)	1,588 (56.80)	241 (37.80)		
Follow up (months)	103.00 (68.00–147.00)	112.50 (74.00–150.00)	71.00 (41.00–111.25)	< 0.001	
Body mass index (kg/m^2^)	29.73 (27.35–33.24)	29.69 (27.36–33.17)	29.94 (27.26–33.70)	0.315	
Body mass index category				0.329	
Overweight (25.00–29.90)	1,793 (52.20)	1,471 (52.60)	322 (50.50)		
Obese (≥ 30)	1,641 (47.80)	1,325 (47.40)	316 (49.50)		
Smoking				< 0.001	
< 100 cigarettes/lifetime	1,783 (51.90)	1,521 (54.40)	262 (41.10)		
≥ 100 cigarettes/lifetime	1,651 (48.10)	1,275 (45.60)	376 (58.90)		
Alcohol consumption				< 0.001	
< 1 drink/day	1,351 (39.30)	1,002 (35.80)	349 (54.70)		
≥ 1 drink/day	2,083 (60.70)	1,794 (64.20)	289 (45.30)		
Diabetes	905 (26.40)	666 (23.80)	239 (62.50)	< 0.001	
Hypertension	1,931 (56.20)	1,473 (52.70)	458 (71.80)	< 0.001	
Laboratory data					
Serum creatinine (µmol/L)	103.43 (95.47–114.04)	103.43 (96.36–113.15)	104.31 (91.94–116.69)	0.811	
eGFR (CKD-EPI; (mL/min/1.73 m^2^)	51.73 (44.94–56.36)	52.55 (46.51–56.81)	46.55 (39.38–53.12)	< 0.001	
Blood urea nitrogen (mmol/L)	5.71 (4.64–7.14)	5.36 (4.64–6.78)	6.43 (5.00–8.21)	< 0.001	
Uric acid (mmol/L)	380.70 (333.10–434.20)	380.70 (327.10–428.30)	392.60 (333.10–452.00)	< 0.001	
Serum albumin (g/L)	43.00 (41.00–44.00)	43.00 (41.00–45.00)	42.00 (39.00–43.00)	< 0.001	
Total cholesterol (mmol/L)	5.04 (4.32–5.74)	5.04 (4.34–5.74)	4.91 (4.11–5.65)	0.005	
Triglycerides (mmol/L)	1.59 (1.06–2.39)	1.58 (1.06–2.39)	1.60 (1.07–2.34)	0.890	
Leisure-time activity					
VPA (min/week)	0.00 (0.00–20.00)	0 (0.00–56.00)	0.00 (0.00–0.00)	< 0.001	
VPA category				< 0.001	
< 10 min/week	2,537 (73.90)	1,989 (71.10)	548 (85.90)		
10–149 min/week	418 (12.20)	363 (13.00)	55 (8.60)		
≥ 150 min/week	479 (13.90)	444 (15.90)	35 (5.50)		
MPA (min/week)	0.00 (0.00–136.50)	0.00 (0.00–135.00)	0.00 (0.00–140.00)	0.056	
MPA category				0.025	
< 10 min/week	1,799 (52.40)	1,428 (51.10)	371 (58.20)		
10–149 min/week	820 (23.90)	707 (25.30)	113 (17.70)		
≥ 150 min/week	815 (23.70)	661 (23.60)	154 (24.10)		
MVPA (min/week)	49.00 (0.00–240.00)	60.00 (0.00–240.00)	0.00 (0.00–180.00)	< 0.001	
MVPA category				< 0.001	
< 10 min/week	1,505 (43.80)	1,157 (41.40)	348 (54.50)		
10–149 min/week	751 (21.90)	640 (22.90)	111 (17.40)		
150–299 min/week	455 (13.20)	392 (14.00)	63 (9.90)		
≥ 300 min/week	723 (21.10)	607 (21.70)	116 (18.20)		

The data are expressed as medians (interquartile ranges) or n (%). eGFR, estimated glomerular filtration rate; CKD-EPI, Chronic Kidney Disease Epidemiology Collaboration; VPA, vigorous physical activity; MPA, moderate physical activity; MVPA, moderate-to-vigorous physical activity.

## Associations of PA with all-cause mortality

**Fig. 2:**
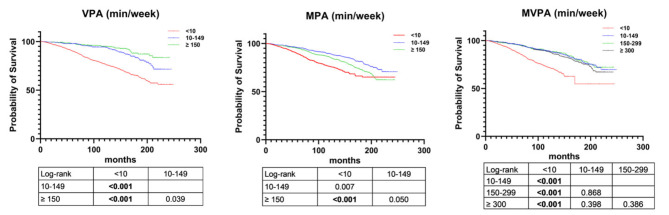
Kaplan–Meier curves of survival according to physical activity.VPA, vigorous physical activity; MPA, moderate physical activity; MVPA, moderate-to-vigorous physical activity

Variations in the VPA and MPA categories were analyzed. The subject age, gender, educational level, smoking status, alcohol consumption, BMI, eGFR, and serum albumin, blood urea nitrogen, serum creatinine, and total cholesterol levels differed significantly across VPA and MPA categories (all *p* < 0.05; Supplementary Tables 1 and 2). The Kaplan–Meier analysis indicated that patients who engaged in < 10 min PA per week had a significantly greater probability of all-cause mortality (log-rank *p* < 0.001; Fig. 2). In contrast, patients who engaged in a minimum of 150 min VPA or 10–149 min MPA weekly had a significantly greater probability of survival (log-rank *p* < 0.001; Fig. 2).

In the univariable Cox proportional hazard analysis, all-cause mortality was associated significantly with the subject age, gender, educational level, smoking status, alcohol consumption, diabetes status, hypertension status, eGFR, and uric acid, serum albumin, serum creatinine, blood urea nitrogen, and total cholesterol levels, as well as VPA. In the multivariable analysis, the subject age, gender, smoking status, alcohol consumption, diabetes status, eGFR, serum albumin level, uric acid level, blood urea nitrogen level, and VPA were identified as independent factors for all-cause mortality (Table 2).

**Table 2:** Variables associated with all-cause mortality during follow up, as determined by Cox proportional hazard analysis

Variable	Univariable analysis		Multivariable analysis	
	HR (95% CI)	*p*	HR (95% CI)	*p*
Age	1.106 (1.096–1.116)	< 0.001	1.097 (1.086–1.108)	< 0.001
Gender	1.588 (1.328–1.899)	< 0.001	0.675 (0.558–0.817)	< 0.001
Education	0.467 (0.398–0.549)	< 0.001		
Smoking	1.672 (1.427–1.957)	< 0.001	1.307 (1.110–1.539)	0.001
Alcohol consumption	0.462 (0.395–0.540)	< 0.001	0.697 (0.592–0.821)	< 0.001
Diabetes	2.095 (1.783–2.462)	< 0.001	1.256 (1.064–1.482)	0.007
Hypertension	2.230 (1.876–2.651)	< 0.001		
eGFR	0.941 (0.934–0.948)	< 0.001	0.985 (0.976–0.994)	0.001
Blood urea nitrogen	1.133 (1.113–1.153)	< 0.001		
Uric acid	1.002 (1.001–1.003)	< 0.001	1.002 (1.001–1.003)	< 0.001
Serum albumin	0.869 (0.851–0.887)	< 0.001	0.916 (0.894–0.939)	< 0.001
Total cholesterol	0.861 (0.798–0.929)	< 0.001		
VPA	0.996 (0.995–0.997)	< 0.001	0.999 (0.998–1.000)	0.024
MPA	1.000 (0.999–1.000)	0.119		
MVPA	0.999 (0.999–0.999)	< 0.001		

HR, hazard ratio; CI, confidence interval; eGFR, estimated glomerular filtration rate; VPA, vigorous physical activity; MPA, moderate physical activity; MVPA, moderate-to-vigorous physical activity.

**Fig. 3:**
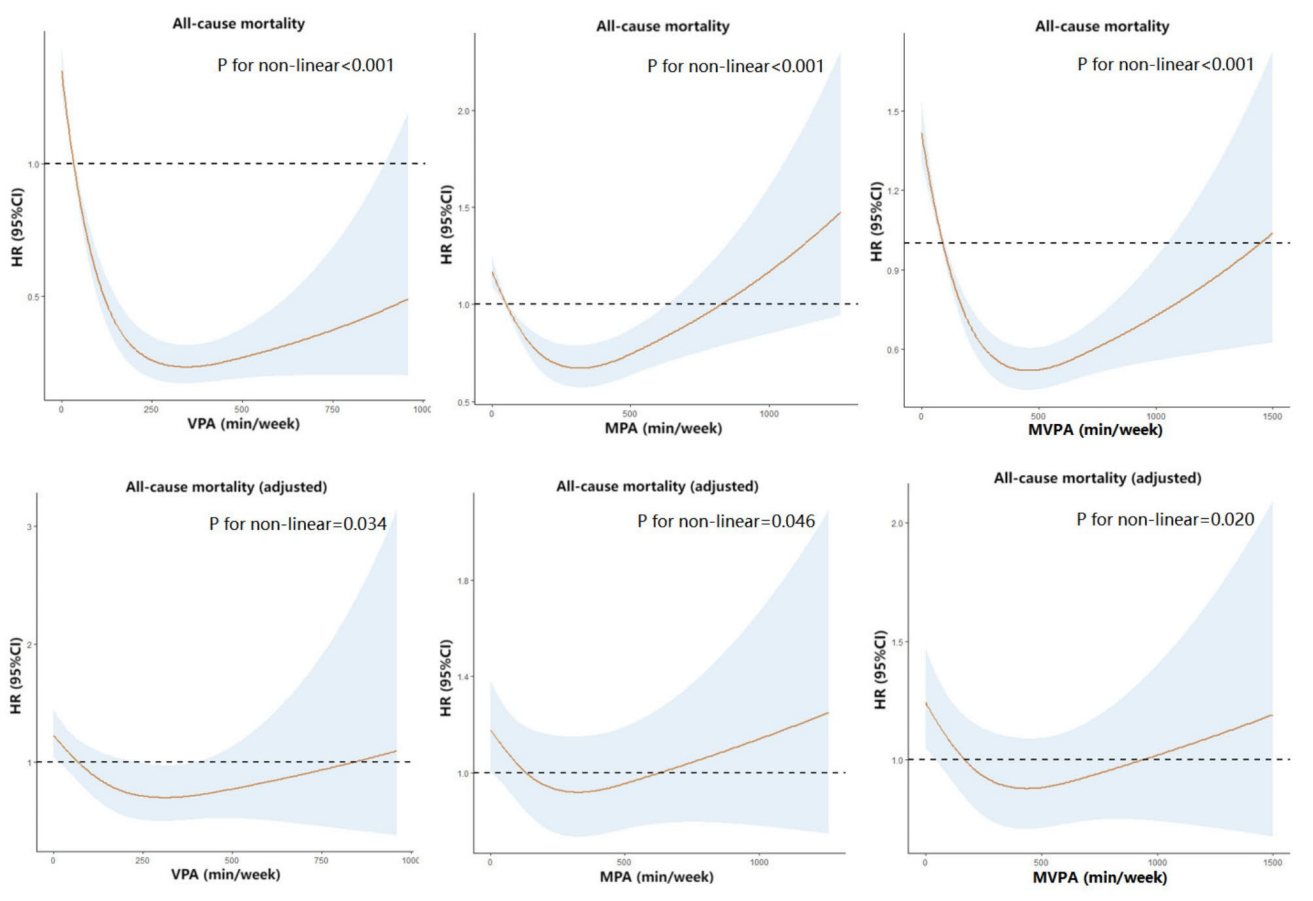
Restricted cubic spline models of survival according to physical activity. The model had three knots at the 10th, 50th, and 90th percentiles and was established using the physical activity duration (min/week) as the scale. VPA, vigorous physical activity; MPA, moderate physical activity; MVPA, moderate-to-vigorous physical activity

In the restricted cubic spline model, VPA, MPA, and MVPA exhibited U-shaped relationships with all-cause mortality (all *p* < 0.05). This relationship persisted after adjustment for subjects’ age, gender, smoking status, alcohol consumption, diabetes status, eGFR, and blood urea nitrogen, uric acid, and serum albumin levels (Fig. 3).

## Appropriate PA for overweight and obese patients with CKD

**Fig. 4:**
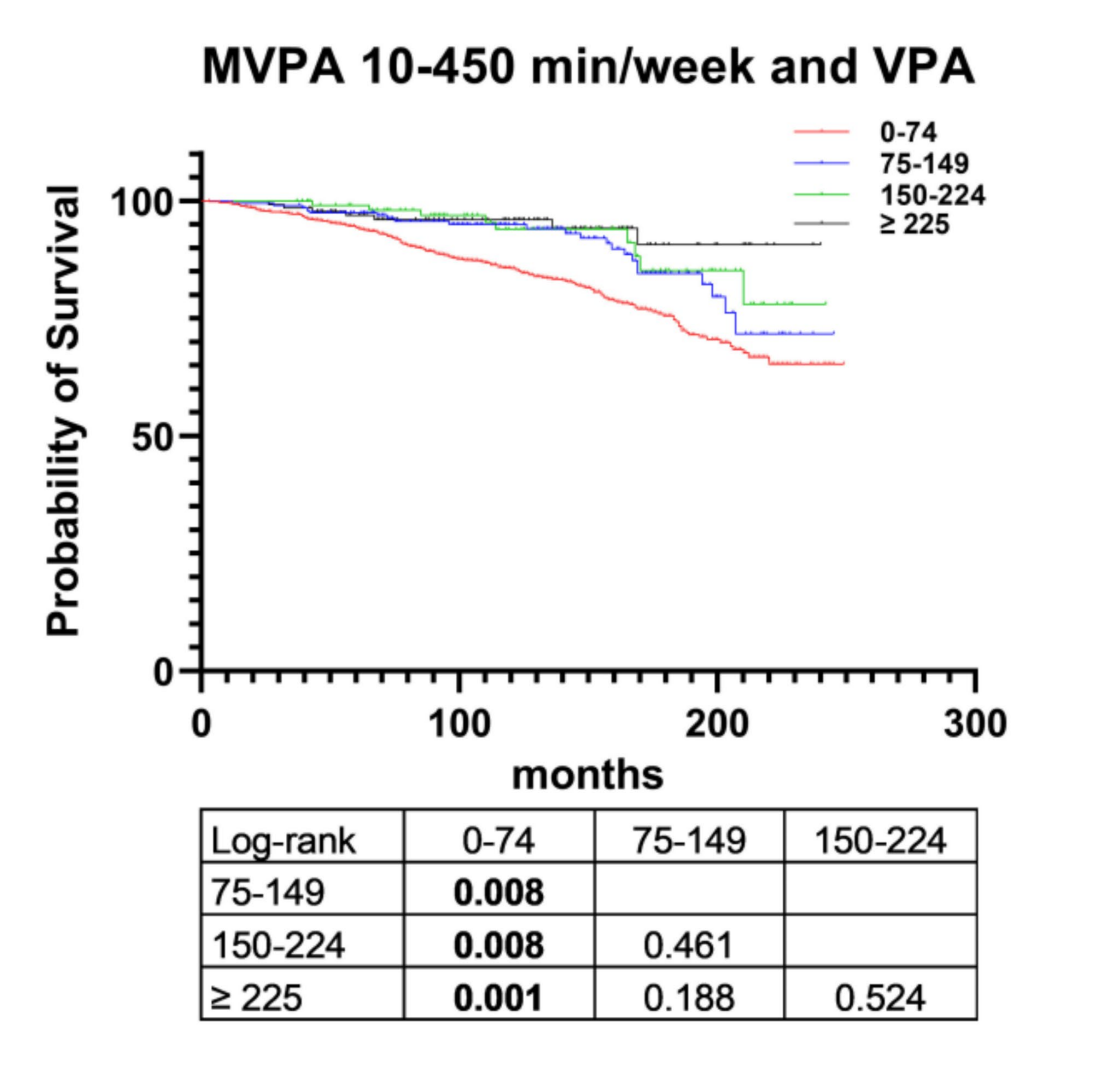
Kaplan-Meier curve of survival for patients who engaged in 10–450 min MVPA/week according to VPA. VPA, vigorous physical activity; MPA, moderate physical activity; MVPA, moderate-to-vigorous physical activity

Patients who engaged in 10–450 min MVPA per week (*n* = 1,505; 78.2% of patients engaging in ≥ 10 min MVPA/week) were further classified into four groups based on their VPA (0–74, 75–149, 150–224, and ≥ 225 min/week). The characteristics of these patients are presented in Supplementary Table 3. All-cause mortality and the median MPA/MVPA ratio decreased with increasing VPA duration. More patients who engaged in ≥ 150 min MPA per week engaged in 0–74 min VPA per week relative to other VPA durations. The Kaplan–Meier curves indicated that higher levels of VPA were associated with reduced all-cause mortality. However, no significant difference was identified between the 150–224 min VPA group and the ≥ 225 min VPA (*p* = 0.283) or 75–149 min VPA (*p* = 0.288) group. Significant differences were identified between 0–74 min VPA and  ≥ 75 min VPA groups (log-rank *p* < 0.01; Fig. 4).

## Discussion

The present study was conducted to examine free-time PA in overweight and obese patients with CKD. It showed that patients who engaged in MVPA had a lower all-cause mortality rate, and that VPA was a predictor of all-cause mortality. Restricted cubic spline models revealed U-shaped relationships of VPA, MPA, and MVPA with all-cause mortality. For patients who engaged in 10–450 min MVPA weekly, a minimum of 75 min VPA per week had significant health benefits.

The median ages of survivors and non-survivors in the present sample were 74 and 60 years, suggest that advanced age is a risk factor for mortality in this population. Age also influenced PA. The Berlin Aging Study II, conducted with subjects aged ≥ 60 years at baseline, revealed a weak negative correlation between age and PA (MET and MPA), but no significant correlation between age and light PA [22]. We can also reasonably expect that older people engage in less VPA. Thus, age is an important confounder in analyses of the effects of PA intensity and duration. Available studies reveal that regular PA is considered a preventive measure against oxidative stress-related diseases, and MPA can promote beneficial effects of physical activity on oxidative stress in elderly subjects [23], but the impact of VPA in elder people is still remin uncertain.

The majority (80%) of subjects included in this study were male; 81.30% of survivors and 74.50% of non-survivors were male, suggesting that being male is associated with a lower risk of mortality in this population. However, males with CKD have been found in other research to have a higher rate of all-cause mortality than females [24]. In the present study, the multivariable Cox proportional hazards analysis supported male gender as a protective factor (0.675; 95% CI, 0.558–0.817), whereas the univariable analysis result was contrary (1.588; 95% CI, 1.328–1.899). We found that age played a key role in this reversal. Given the sample size, this could be the result of data bias or unidentified associations. The present results for PA support the previous finding that males are more likely than females to engage in PA, especially VPA [25].

In the present sample, non-survivors were less likely than survivors to have higher levels of education (37.80% vs. 56.80%). A recent meta-analysis demonstrated that each year of education reduces the mortality risk by 1.9% (95% uncertainty interval, 1.8–2.0%) in all ages, even 2.9% (95% uncertainty interval, 2.8–3.0%) in individuals aged 18–49 years [26].

Smoking was more prevalent among non-survivors than among survivors in this study (58.90% vs. 45.60%). The effect of smoking on mortality is known. Recreational PA was also found to have reverse correlation with smoking behavior (*p* < 0.001; odds ratio = 0.729; 95% Cl, 0.639–0.832) [27]. Thus, PA can be used as a tobacco use control measure, which may help to reduce mortality.

Alcohol consumption was more prevalent among non-survivors than among survivors in this study. However, this finding should not be interpreted as indicating that changes in individuals’ alcohol consumption can reduce all-cause mortality. The Global Burden of Alcohol study showed that each increment (8 g pure alcohol) in alcohol consumption is associated with an increased risk of loss of disability-adjusted life-years [28]. The result may be attributed to the cross-sectional nature of the study, self-underreporting of alcohol consumption [29], and/or confounding.

Diabetes and hypertension were more prevalent among non-survivors than among survivors in this study, which may reasonably be attributed to the older age of the former. Numerous studies have demonstrated the benefits of PA in patients with diabetes and/or hypertension, including the reduction of the systolic blood pressure, the incidence of mortality [30], insulin resistance [31], and the incidence of complications [32–34]. A volume of ≥ 150 min MPA per week was recommended for patients with diabetes or hypertension, even a volume of 90 min VPA per week was recommended for patients with diabetes to reinforcement benifits [35]. Meanwhile, we should aware that additional safety considerations are needed for such patients. When planing exercise, the patient should be examined in greater detail (e.g. cardiopulmonary exercise testing) and treated accordingly. In patients with retinopathy, VPA, activities with jumps and the Valsalva manoeuvre should be avoided. Patients with peripheral neuropathy or peripheral vascular disease on feet should always be inspected and avoid weight-bearing exercise. Measurements of blood glucose concentrations and blood pressure should be made before, during and at the end of each session. When a hypoglycaemic episode has occurred during the last 24 h or resting systolic blood pressure > 160 mmHg, the exercise should be postponed. During the exercise intervention, if systolic blood pressue rises to > 250 mmHg or diastolic blood pressure > 115 mmHg, the training session should be terminated and the patients should visit their doctor.

The eGFR, blood urea nitrogen, uric acid, serum creatinine levels, and serum albumin were worse among non-survivors than among survivors in this study, indicating lower renal function and nutritional status of non-survivor which was consistent with the trend of VPA or MVPA. Age as an independent risk factor for decreased renal function, is higher in non-survivors. Inaddition, evidence from meta-analysis revealed that PA can improves eGFR by 2.16 ml/min/1.73m^2^ in non-dialysis CKD patients [36, 37]. A longitudinal cohort study also found each 60-minute increment in PA weekly was associated with a 0.5% slower decline per year in eGFR [38]. This emphasizes the importance of PA for CKD patients again.

In the present sample, non-survivors spent less time engaging in MPA than did survivors, suggesting that PA reduces mortality. Similarly, the Kaplan–Meier analysis indicated that mortality was lower among subjects who engaged in MPA than among those who did not (log-rank *p* < 0.001). Consensus has been reached that inactivity is unhealthy, and that even low levels of activity are preferable; the use of MPA to achieve this goal has been widely studied and applied. MPA has numerous forms [20], and is thus applicable for large proportions of population. Appropriate MPA can reduce a variety of health risks, such as those of mortality, cardiovascular disease, stroke, high blood pressure, and type 2 diabetes [39–43]. It also improves glycemic control, regulates the body weight, and improves sleep quality, among other benefits [44–46]. When it comes to increasing the intensity of exercise, indivisuals often worry about the risk of injury, cardiovascular accidents, and effects on underlying diseases. Compaired to sedentary, any activity may raise the risk of injury which the musculoskeletal injury is the most. Studies had found MPA had low rates of musculoskeletal injuries, some reports have reported that injuries were more common during early stages of overload and adaptation [47]. As for cardiovascular accidents, it should be emphasized that a sedentary lifestyle is associated with much higher risk of adverse cardiac outcomes and all-cause mortality than any level of PA [48]. Underlying diseases such as diabetes and hypertension have been mentioned foreword, PA recommendations for various diseases were being studied. We can expect that most worries can be greatly improved through proper exercise arrangements.

Consistent with the findings for MPA, non-survivors spent less time than survivors engaging in VPA, suggesting that PA reduces mortality. The multivariable Cox proportional hazards analysis indicated that VPA (0.024; 95% CI, 0.998–1.000) was an independent factor for all-cause mortality. In support of these findings, higher levels of VPA within MVPA have been associated with lower all-cause mortality [49, 50]. VPA has been shown to significantly improve cardiorespiratory fitness and certain cardiometabolic risk factors [51–53]. Short bouts of VPA can improve markers of insulin sensitivity and glucose regulation [54]. In addition, VPA boosts immune surveillance and reduces systemic inflammatory activity [55]. In a cohort of 813 patients with nonalcoholic fatty liver, those who engaged regularly in VPA had significantly less steatohepatitis and advanced fibrosis than those who participated in MPA [56]. In recent years, high-intensity interval training has been proposed as a time-efficient exercise protocol to improve health. In a cohort of overweight/obese middle-aged men, low-volume high-intensity interval training and higher-volume moderate-intensity continuous training were associated with similar enjoyment levels and high (> 90%) unsupervised adherence rates [57]. A meta-analysis of 10 studies found no difference in rates of adherence to unsupervised high-intensity interval training and moderate-intensity interventions [58]. From a clinical perspective, VPA is not hard to maintain for individuals with obesity, which may spent less time and gain extra benifits. However, VPA also has disadvantages. Evidence suggests that engagement in extreme-intensity PA increases the risk of acute cardiac events [59]. Cardiac concerns include sudden cardiac death, myocardial fibrosis, pathological remodeling, and PA-induced arrhythmias [60]. Furthermore, the findings of a recent study suggest that the exercise intensity, rather than volume, is associated with more coronary artery calcification (CAC) and plaque progression in senior endurance sportsmen [61]. This observation can be explained by homeostatic imbalances, such as alternative coronary hemodynamics and increased levels of inflammatory cytokines, parathyroid hormone, and reactive oxygen species, and the influence of testosterone [62]. Although CAC is generally linked to a higher risk of adverse cardiac event occurrence in the general population, it may not be an appropriate measure for endurance athletes. Other factors such as age， underlying diseases and physical conditions can also affect the safety and benefits of VPA.

These considerations highlight the importance of setting an upper limit on the PA intensity, especially for the growing population of overweight and obese patients with CKD. The present study also showed that the relationship between VPA and all-cause mortality was non-linear after adjustment, but the sample size limited the exploration of a cut-off point for VPA and sufficient adjustment for confounding factors.

VPA and MPA are ideal PA methods, with mutual influences that can be attributed to individual limitations of physical capacity. The Kaplan–Meier analysis conducted in this study indicated that 10–149 min MPA weekly was associated with a significantly greater probability of survival, which is inconsistent with existing guidelines. Further analysis revealed that the median MPA/MVPA ratio decreased with increasing VPA duration, and that more patients who engaged in MPA for ≥ 150 min per week engaged in 0–74 min VPA relative to other VPA duration, partly explaining the results of the initial analysis. Further research on combinations of VPA and MPA are warranted to guide the personalization of interventions.

The WHO, KDIGO, AACE/ACE and ACC/AHA guidelines all agree on the recommendation of ≥ 150 min MPA per week, but recommendations for VPA vary. The present findings suggest that a minimum of 75 min VPA per week, similar to the WHO recommendation, would be beneficial.

### Study limitations and future directions

The present study has several limitations. First, residual confounding factors, such as weight loss, could not be completely ruled out, despite adjustment for multiple impactful factors at baseline. Second, the PA data were based on self-reporting at a single timepoint, which could have led to measurement inaccuracy. Third, the small size of the study population limited the ability to conduct a detailed analysis of each PA type, and to fully evaluate the potential drawbacks of higher VPA levels. We recommend further study of CKD, obesity, and VPA using the UK Biobank, a large population-based data resource with more than 500,000 participants that provides extensive follow-up data (e.g., on weight changes) and detailed outcome (e.g., ESRD) records. Such research could include the assessment of the disadvantages of VPA to identify an appropriate exercise balance, and the exploration of associations between VPA and cardiovascular disease and joint lesions.

## Conclusions

Longer durations of VPA are associated with less all-cause mortality in overweight and obese patients with CKD. Clinicians should be aware of the benefits associated with VPA when providing consultation to such patients, and a minimum of 75 min VPA weekly could serve as a baseline for advice and public health interventions.

## Electronic supplementary material

Below is the link to the electronic supplementary material.

Supplementary Material 1

## Data Availability

The datasets generated during and/or analysed during the current study are available in the NHANES repository, https://wwwn.cdc.gov/nchs/nhanes/.

## Abbreviations

CKD: Chronic kidney disease
PA: Physical activity
BMI: Body mass index
eGFR: Estimated glomerular filtration rate
ESRD: End-stage renal disease
WHO: World Health Organization
MPA: Moderate physical activity
VPA: Vigorous physical activity
KDIGO: Kidney Disease: Improving Global Outcomes
AACE/ACE: American Association of Clinical Endocrinologists/American College of Endocrinology
ACC/AHA: American College of Cardiology/American Heart Association
NHANES: National Health and Nutrition Examination Survey
NDI: National Death Index
NCHS: National Center for Health Statistics
CDC: Centers for Disease Control and Prevention
MET: Metabolic equivalent
MVPA: Moderate-to-vigorous physical activity
CKD-EPI: Chronic Kidney Disease Epidemiology Collaboration
