# Supplementary material for: Exercise intensity and mortality in overweight and obese patients with chronic kidney disease: longitudinal analysis (1999–2016)
Source: BMC Public Health. 2024 Oct 31;24:3020. doi: 10.1186/s12889-024-20498-6 (PMC11529189; doi:10.1186/s12889-024-20498-6)
Supplement: Supplementary file 1 — Supplementary Material 1 [file 12889_2024_20498_MOESM1_ESM.docx]

Supplementary Material

Supplementary material

**Supplementary Table 1** Patient characteristics by VPA category

| **Characteristic** | **< 10 min/week**  **(*n* = 2,537)** | **10–149 min/week**  **(*n* = 418)** | **≥ 150 min/week**  **(*n* = 479)** | ***p*** |
| --- | --- | --- | --- | --- |
| Clinical characteristics | | | | |
| Age (years) | 64 (55–74) | 50 (40–63) | 50 (40–61) | <0.001 |
| Gender (male) | 1,915 (75.50) | 383 (91.60) | 449 (93.70) | <0.001 |
| Race/ethnicity |  |  |  | 0.482 |
| Non-Hispanic white | 1,144 (45.10) | 194 (46.40) | 204 (42.60) |  |
| Other | 1,393 (54.90) | 224 (53.60) | 275 (57.40) |  |
| Education |  |  |  | <0.001 |
| High school graduation or less | 1,369 (54.00) | 120 (28.70) | 116 (24.20) |  |
| College graduation or more | 1,168 (46.00) | 298 (71.30) | 363 (75.80) |  |
| Follow up (months) | 94.00 (63.00–132.00) | 154.50 (96.00–179.00) | 125.00 (80.00–171.00) | <0.001 |
| Body mass index (kg/m^2^) | 30.00 (27.56–33.82) | 29.14 (27.06–31.95) | 28.83 (26.81–31.64) | <0.001 |
| Body mass index category |  |  |  | <0.001 |
| Overweight (25.00–29.90) | 1,254 (49.40) | 238 (56.90) | 301 (62.80) |  |
| Obese (≥ 30） | 1,283 (50.60) | 180 (43.10) | 178 (37.20) |  |
| Smoking |  |  |  | <0.001 |
| < 100 cigarettes/lifetime | 1,209 (47.70) | 263 (62.90) | 311 (64.90) |  |
| ≥ 100 cigarettes/lifetime | 1,328 (52.30) | 155 (37.10) | 168 (35.10) |  |
| Alcohol |  |  |  | <0.001 |
| < 1 drink/day | 1,100 (43.40) | 117 (28.00) | 134 (28.00) |  |
| ≥ 1 drink/day | 1,437 (56.60) | 301 (72.00) | 345 (72.00) |  |
| Diabetes | 787 (31.00) | 65 (15.60) | 53 (11.10) | <0.001 |
| Hypertension | 1,578 (62.20) | 174 (41.60) | 179 (37.40) | <0.001 |
| Mortality | 548 (21.60) | 55 (13.20) | 35 (7.30) | <0.001 |
| Laboratory data | | | | |
| Serum creatinine (μmol/L) | 101.66 (93.70–113.15) | 106.08 (97.20–114.92) | 106.08 (97.24–114.92) | <0.001 |
| eGFR  (CKD-EPI; mL/min/1.73 m^2^) | 51.12 (43.70–56.12) | 53.50 (47.60–56.97) | 53.33 (48.29–56.66) | <0.001 |
| Blood urea nitrogen (mmol/L) | 5.71 (4.64–7.50) | 5.00 (4.28–6.08) | 5.36 (4.28–6.43) | <0.001 |
| Uric acid (mmol/L) | 380.70 (333.10–434.20) | 374.70 (327.10–428.30) | 374.70 (327.10–422.30) | 0.059 |
| Serum albumin (g/L) | 42.00 (40.00–44.00) | 43.00 (41.00–45.00) | 43.00 (42.00–45.00) | <0.001 |
| Total cholesterol (mmol/L) | 4.99 (4.24–5.73) | 5.21 (4.58–5.82) | 5.09 (4.42–5.74) | <0.001 |
| Triglycerides (mmol/L) | 1.64 (1.10–2.44) | 1.50 (1.00–2.26) | 1.43 (0.96–2.17) | <0.001 |

The data are expressed as medians (interquartile ranges) or *n* (%). VPA, vigorous physical activity; eGFR, estimated glomerular filtration rate; CKD-EPI, Chronic Kidney Disease Epidemiology Collaboration.

**Supplementary Table 2** Patient characteristics by MPA category

| **Characteristic** | **< 10 min/week**  **(*n* = 1,799)** | **10–149 min/week**  **(*n* = 820)** | **≥ 150 min/week**  **(*n* = 815)** | ***p*** |
| --- | --- | --- | --- | --- |
| Clinical characteristic | | | | |
| Age (years) | 63 (52–74) | 60.00 (48–68) | 61.00 (49–70) | <0.001 |
| Gender (male) | 1,364 (75.80) | 665 (81.10) | 718 (88.10) | <0.001 |
| Rac/ethnicity |  |  |  | <0.001 |
| Non-Hispanic white | 750 (41.70) | 385 (47.00) | 407 (49.90) |  |
| Other | 1,049 (58.30) | 435 (53.00) | 408 (50.10) |  |
| Education |  |  |  | <0.001 |
| High school graduation or less | 996 (55.40) | 295 (36.00) | 314 (38.50) |  |
| College graduation or more | 803 (44.6) | 525 (64.00) | 501 (61.50) |  |
| Follow up (months) | 92.00 (61.00–128.00) | 124.00 (76.00–170.00) | 121.00 (77.00–167.00) | <0.001 |
| Body mass index (kg/m^2^) | 30.00 (27.53–34.06) | 29.81 (27.34–32.76) | 29.20 (27.01–32.28) | <0.001 |
| Body mass index category |  |  |  | <0.001 |
| Overweight (25.00–29.90) | 894 (49.70) | 421 (51.30) | 478 (58.70) |  |
| Obese (≥ 30） | 905 (50.30) | 399 (48.70) | 337 (41.30) |  |
| Smoking |  |  |  | 0.006 |
| < 100 cigarettes/lifetime | 897 (49.90) | 464 (56.60) | 422 (51.80) |  |
| ≥ 100 cigarettes/lifetime | 902 (50.10) | 356 (43.40) | 393 (48.20) |  |
| Alcohol consumption |  |  |  | <0.001 |
| < 1 drink/day | 804 (44.70) | 290 (35.40) | 257 (31.50) |  |
| ≥ 1 drink/day | 995 (55.30) | 530 (64.60) | 558 (68.50) |  |
| Diabetes | 543 (30.20) | 196 (23.90) | 166 (20.40) | <0.001 |
| Hypertension | 1,062 (59.00) | 436 (53.20) | 433 (53.10) | 0.002 |
| Mortality | 371 (20.60) | 113 (13.80) | 154 (18.90) | <0.001 |
| Laboratory data | | | | |
| Serum creatinine (μmol/L) | 103.43 (94.59–114.92) | 104.31 (97.24–112.27) | 101.66 (95.47–112.27) | 0.018 |
| eGFR  (CKD-EPI; mL/min/1.73 m^2^) | 50.87 (43.39–56.03) | 52.41 (46.35–56.66) | 52.54 (46.75–56.56) | <0.001 |
| Blood urea nitrogen (mmol/L) | 5.71 (4.64–7.50) | 5.36 (4.64–6.78) | 5.36 (4.64–6.78) | <0.001 |
| Uric acid (mmol/L) | 380.70(333.10–434.20) | 374.70 (327.10–434.20) | 380.70 (327.10–428.30) | 0.229 |
| Serum albumin (g/L) | 42.00 (40.00–44.00) | 43.00 (41.00–45.00) | 43.00 (41.00–45.00) | <0.001 |
| Total cholesterol (mmol/L) | 4.97 (4.22–5.66) | 5.17 (4.44–5.82) | 5.07 (4.40–5.79) | <0.001 |
| Triglycerides (mmol/L) | 1.63 (1.08–2.43) | 1.58 (1.07–2.33) | 1.51 (1.03–2.34) | 0.169 |

The data are expressed as medians (interquartile ranges) or *n* (%). MPA, moderate physical activity; eGFR, estimated glomerular filtration rate; CKD-EPI, Chronic Kidney Disease Epidemiology Collaboration.

**Supplementary Table 3** Characteristics of patients who engaged in 10–450 min MVPA per week by VPA category (the values are expressed in median [interquartile range] or n [%]).

| **Characteristic** | **0–74 min/week (*n* = 1063)** | **75–149 min/week (*n* = 200)** | **150–224 min/week (*n* = 108)** | **≥ 225 min/week *(n* = 134)** |
| --- | --- | --- | --- | --- |
| Clinical characteristics | | | | |
| Age (years) | 56 (46–65) | 51 (40–63) | 50 (42–59.50) | 47.(37–58) |
| Gender (male) | 852 (80.20) | 182 (91.00) | 99 (91.70) | 123 (91.80) |
| Race/ethnicity |  |  |  |  |
| Non-Hispanic white | 285 (50.40) | 116 (58.00) | 58 (53.70) | 79 (59.00) |
| Other | 280 (49.60) | 84 (42.00) | 50 (46.30) | 55 (41.00) |
| Education |  |  |  |  |
| ≤High school graduation | 445 (41.90) | 54 (27.00) | 24 (22.20) | 31 (23.10) |
| ≥College graduation | 618 (58.10) | 146 (73.00) | 84 (78.80) | 103 (76.90) |
| Follow up (months) | 119.00 (75.00–167.00) | 136.50 (76.25–178.75) | 125 (91.25–173.75) | 119.00 (69.75–164.25) |
| Body mass index (kg/m^2^) | 29.58 (27.34–33.07) | 29.38 (27.00–32.04) | 28.96 (27.01–32.27) | 28.70 (26.87–30.90) |
| Body mass index category |  |  |  |  |
| Overweight (25.0–29.9) | 571 (53.70) | 109 (54.50) | 66 (61.10) | 88 (65.70) |
| Obese (≥ 30) | 492 (46.30) | 91 (45.50) | 42 (38.90) | 46 (34.30) |
| Smoking |  |  |  |  |
| < 100 cigarettes/life | 570 (53.60) | 125 (62.50) | 67 (62.00) | 92 (68.70) |
| ≥ 100 cigarettes/life | 493 (46.40) | 75 (37.50) | 41 (38.00) | 42 (31.30) |
| Alcohol, consumption |  |  |  |  |
| < 1 drink/day | 396 (37.30) | 54 (27.00) | 28 (25.90) | 36 (26.90) |
| ≥ 1 drink/day | 667 (62.70) | 146 (73.00) | 80 (74.10) | 98 (73.10) |
| Diabetes | 269 (25.30) | 34 (17.00) | 12 (11.10) | 13 (9.70) |
| Hypertension | 596 (56.10) | 93 (46.50) | 41 (38.00) | 42 (31.30) |
| Mortality | 189 (17.80) | 22 (11.00) | 9 (8.30) | 7 (5.20) |
| Laboratory data | | | | |
| Serum creatinine (μmol/L) | 100.78 (95.47–110.50) | 106.08 (97.24–114.92) | 106.08 (98.12–111.83) | 106.08 (99.89–114.92) |
| eGFR  (CKD-EPI; mL/min/1.73 m^2^) | 52.42 (46.09–56.82) | 53.19 (48.07–56.73) | 53.04 (48.18–55.69) | 53.11 (48.56–57.07) |
| Blood urea nitrogen (mmol/L) | 5.36 (4.64–6.78) | 5.00 (4.28–6.07) | 5.36 (4.64–6.43) | 5.36 (4.64–6.43) |
| Uric acid (mmol/L) | 380.70 (333.10–434.20) | 380.70 (333.10–434.20) | 386.60 (345.00–428.30) | 362.80 (313.73–428.30) |
| Serum albumin (g/L) | 41.00 (43.00–45.00) | 43.00 (41.00–45.00) | 43.00 (41.25–45.00) | 43.00 (42.00–46.00) |
| Total cholesterol (mmol/L) | 5.15 (4.42–5.84) | 5.30 (4.55–5.96) | 5.26 (4.53–5.94) | 5.09 (4.37–5.66) |
| Triglycerides (mmol/L) | 1.60 (1.10–2.36) | 1.46 (0.96–2.35) | 1.51 (1.03–2.34) | 1.43 (0.97–2.11) |
| Leisure-time activity | | | | |
| VPA (min/week) | 0.00 (0.00–0.00) | 120.00 (90.00–121.33) | 180 (175.88–182.00) | 270.92 (240.00–360.00) |
| MPA (min/week) | 112.00 (56.00–210.00) | 60.00 (0.00–139.13) | 65.92 (0.00–150.00) | 0.00 (0.00–64.75) |
| MVPA (min/week) | 120.00 (60.00–210.00) | 176.75 (120.00–259.46) | 245.00 (180.00–334.83) | 353.50 (271.38–375.00) |
| Median MPA/MVPA ratio | 0.93 | 0.34 | 0.27 | 0.00 |

The data are expressed as medians (interquartile ranges) or *n* (%). MVPA, moderate-to-vigorous physical activity; VPA, vigorous physical activity; eGFR, estimated glomerular filtration rate; CKD-EPI, Chronic Kidney Disease Epidemiology Collaboration.
